# The Influence of Interpersonal Behaviors and Population Density on Grip Strength of Elderly People: An Analysis of the Direct vs. Indirect Effects via Social Participation

**DOI:** 10.3389/fpubh.2021.755695

**Published:** 2021-12-10

**Authors:** Haibo Lin, Haijun Ren

**Affiliations:** ^1^Business School, Dalian University of Foreign Languages, Dalian, China; ^2^Department of General Surgery, Dalian Municipal Friendship Hospital, Dalian, China

**Keywords:** health of elderly people, grip strength, community differences, interpersonal environment, social participant, gender differences

## Abstract

The impact of social participation (SP) on the health of the elderly has been widely recognized, and urban-rural differences in social participation have attracted attention. However, few studies discussed the impact of social participation on specific health indicators and the further subdivision of urban-rural differences. This research aims to use the dimensions of interpersonal behaviors and population density rather than simple urban-rural distinctions to justify community differences and compare these differences' direct and indirect effects on grip strength. This study used 15,871 respondents aged over 50 years from the China Health and Retirement Longitudinal Study (CHARLS). An SEM (Structural Equation Modeling) analysis was used to explore the joint effect of interpersonal behavior and population density on social participation and the consequent impact on changes in grip strength and compare the differences among different genders, ages, wealth levels, and family relationships. The results indicated that community differences characterized by interpersonal behavior and population density have direct effects on grip strength and indirect effects on it through social participation. The conclusion is that the frequency of social activities, such as mah-jong and dancing in the Metropolitan Fringe and county-level cities is higher than that in Metropolitan centers. The high frequency of these activities has a positive and indirect impact on grip strength, and community differences have a more significant impact on women's social participation than men. However, the direct effect of community differences as defined by interpersonal communication and population density on grip strength is greater than the indirect effect of other factors through social participation.

## Introduction

Grip strength is an essential indicator of muscle ability (1–6) and weakness (1, 7, 8). Because of its ease of measurement and stability (9), grip strength can be used as an essential comparable biomarker for the standardized evaluation of human body functions (10). Increasingly, grip strength is becoming a major feature of studies on geriatric health. Such as Sayer and Kirkwood (10) said: “An intriguing implication is that grip strength might act as a biomarker of aging across the life course. This is not a new idea, but findings from Prospective Urban Rural Epidemiology PURE add support. Loss of grip strength is unlikely to lie on a single final common pathway for the adverse effects of aging. However, it might be an excellent marker of underlying aging processes?” Grip strength is directly related to cardiovascular disease (11), Alzheimer's disease (7), cognitive impairment (12), and the mortality rate (10, 13). However, the analysis of roles of factors affecting grip strength has not been detailed enough, and the conclusions are not sufficiently clear (14). It is generally believed that the influencing factors of grip strength include age, physical activity (PA), and social activity (SA) (15, 16). Of course, some studies discussed social status, economic conditions, education, and other factors that can affect grip strength through PA and SP (17, 18). However, studies with larger samples, examine the mechanisms of these effects and draw more precise conclusions are needed.

According to the theoretical framework of social ecology, the environment (broadly defined), behavior patterns, and health status interact with each other. Some studies have emphasized the importance of the infrastructural environment (19, 20). This article suggests that the difference between urban and rural areas in China is not only the population density but also the interpersonal habits, Differences in behavior patterns in communities in China are more likely to be the key factors that affect SP, which is considered to be very relevant to the health of elderly people. Accordingly, based on the analysis of the SA of elderly residents of urban and rural areas, this article proposed that the agricultural social customs (behavior patterns) and population density (environment) are important factors that affect the health of elderly people. In other words, community density and behavior affect the health (both physical and psychological) of elderly people through social participation.

In this study, based on a large sample from the China Health and Retirement Longitudinal Study (CHARLS), which was completed by Peking University, this article constructs a conceptual model of community differences, social activities, and change in grip strength through a structural equation model. This article aimed to reveal the joint effect of community differences and lifestyle habits on social participation and the consequent effect on changes in grip strength and to compare the differences among people with different genders, ages, wealth levels, and family relationships. Therefore, (1) This paper built an association model based on communication style and specific biomarkers and used a large sample size to test this relationship. (2) This article expected that a comparison of the intermediate and direct effects could show that non-interventional exogenous variables such as age and gender on grip strength will be partially endogenous (intervenable), rather than just controlling variables. (3) This paper proposed that it is not only the differences between urban and rural areas (in a broad geographical sense) but also the different population densities and cultural backgrounds of residential areas that should be used to classify communities here. In assessing community differences, this article scored types of communities differently than in the original questionnaire. By rearranging the scoring structure for different types of communities, this article revealed the characteristics of different urban communities and rural elderly SA in China and the impact of these differences on grip strength. (4) When discussing the relationship between social activities and grip strength, this article extended the measurable range of social activities. This article also included the occasional care of parents or grandchildren within the scope of active social activities.

## Literature Review and Hypothesis

### Grip Strength as a Biomarker of the Health of Elderly People

Grip strength is regarded as one of the indicators of the health of elderly people. The health of elderly people is divided into the following two categories (21): overall health (22) and specific signs (biomarkers). Different research strategies can also be divided into different categories, including multiple linear modeling with factor and mechanism analysis involving intermediate variables. Of course, there have been some recent studies based on longitudinal data. Specific analyses of the influencing factors of grip strength and muscle atrophy include (1) the impact of PA on grip strength (23, 24); (2) the impact of SA on grip strength (17, 25); (3) the influence of other individuals' heterogeneity on grip strength, the most important of which is age (26, 27); and (4) the relationship between medical indicators and physiological mechanisms, including PA, nutrient intake (protein), oxidative stress, inflammation, and hormone changes, and more systematic therapeutic interventions (14). High levels of serum interleukin (IL)-6, C-reactive protein (CRP), and α1-antichymotrypsin (ACT) are associated with decreased muscle strength or muscle mass (muscular sarcopenia) (28).

### Relationship Between Physical Activity and Grip Strength

The relationship between PA and grip strength was generally investigated in comprehensive studies of a series of health indicators as well as correlation studies (29).

The effect of PA on grip strength is not very clear, and some studies have shown that physical exercise can improve grip strength in middle-aged elderly, and oldest-old groups (23, 30–33). There are fewer studies on the mechanism of this effect. Celis-Morales et al. have argued that grip strength has a moderating effect on the relationship between PA and mortality rate (34). In the oldest-old group, physical exercise has no relationship with improved muscle capacity (24, 30). However, it is generally believed that even if there is a connection between PA and grip strength (33), especially among older women (24), such a relationship would be small or not very significant (35).

Based on the conclusions of some studies with larger samples in the above literature, we propose Hypothesis HA: moderate PA has a positive relationship with grip strength.

### Social Activities and Grip Strength

There are two lines of study of the relationship between SA and grip strength. First, social interaction directly affects health (broadly defined). There are definite conclusions about the impact of social interaction on mental and physical health. However, the positive and negative impacts need to be analyzed based on the specific SA (36, 37). For example, optimism and positivity are always good for health, and in general, frequent interpersonal activities can affect physical conditions. SA has been divided into categories, such as general social connections (38, 39) and semiformal social assistance and formal community support (volunteering) (40), and then discussed with the mediating role of community solidarity or community support (22). However, is the relation between social interaction and grip strength positive or negative regarding specific physical functions? In particular, such discussions have rarely been conducted based on examining different age groups within the elderly population. Some studies have emphasized personal income (25) and indicated that social participation could promote PA and stimulate hormone secretion (41). Theoretical support for the impact of social participation on grip strength suggests that social interaction promotes the secretion hormones, including sex hormones, adrenal hormones, and thyroid hormones (14). Therefore, stimulating the secretion of these hormones would alter muscle function.

The second line of study examines the effect of SA on PA and therefore, on physical health. Socializing online and participating in groups promote PA and self-rated health (36, 42–44). Many studies also seem to be too broad and fail to provide evidence of intermediate mechanisms. For example, Yorke et al. (45) reported a significant relationship between delayed retirement (assessed in a treatment group that differed in terms of their social participation from a peer control group that retired at the expected age) and muscle atrophy as assessed by grip strength. Nevertheless, the authors did not analyze the mechanism of delayed retirement.

Since it discusses a specific health index rather than a general concept of health, this study must find evidence that SA affects grip strength through PA. Studies have discussed the impact of SA and PA on cognitive function to a similar extent (46). Suppose PA is related to cognitive function and neural organization because muscle strength is also related to nerve tissue (47). In that case, it can be concluded that PA and grip strength may be related. However, this is still too general.

Because the mechanism of the relationship among SA, PA, and grip strength is not very clear, and because engagement in boring activities causes depression (43) (due to the secretion of hormones that are negative for health) (42), this article proposes hypothesis HB: positive types of SA ameliorate the decline in the grip strength of elderly people.

### Factors That Affect Both Social Activities and Grip Strength

Many factors affect the SA of elderly people. Here, we focus on the factors that affect both SA and grip strength to compare their direct effects and the indirect effects through SA on grip strength. This approach will allow for a better decomposition of the effects. The community environment, family relations, economic status, age, and gender are of particular interest.

#### Community Differences

The community environment includes the physical environment and social environment (interpersonal environment is the primary representation). China is an urban-rural dual social form. It is recognized that there are significant differences between urban and rural elderly communities in China. It is impossible to analyze the urban-rural differences of Chinese elderly life using the analyzing framework of the United States (48–50). However, in addition to these differences, we note that the community environment that has advanced facilities does not necessarily positively impact social participation. Neighborhood relationship culture has attracted attention recently (51). In China, generally, the elderly in the city centers does not have more SA than the elderly communities at the junction of urban and rural areas (urban fringe). SA based on city center communities are more difficult to organize than those at the junction of urban, rural areas and towns. For example, mah-jongg games greatly increase social activities, while big cities are separated from rural areas (rural culture) for a long time Therefore, they are less keen on such activities, like square dance, which has the same characteristics (12, 52). Therefore, the community environment conducive to social communication can be greatly improved by not completely relying on the existing organizational forms and facility construction (53). So we propose hypothesis HC: The combination of population density and neighborhood relations is positively correlated with SP.

#### Wealth

By analyzing health data for elderly Europeans, Jancova-Vseteckova et al. (17) found that education, professional class, income, and wealth had a predictive effect on male grip strength, while only education and wealth had a predictive effect on female grip strength. However, they provided no further analysis of more complicated impact mechanisms; while they showed that wealth affected grip strength, there was no explanation for the effect. Regarding social status, studies have also reported its relationship with grip strength (18), but it is even harder to find logical analyses of this relationship. Therefore, hypothesis HD is proposed: living standard is positively related to grip strength.

### Structural Equation Model

**Figure 1:**
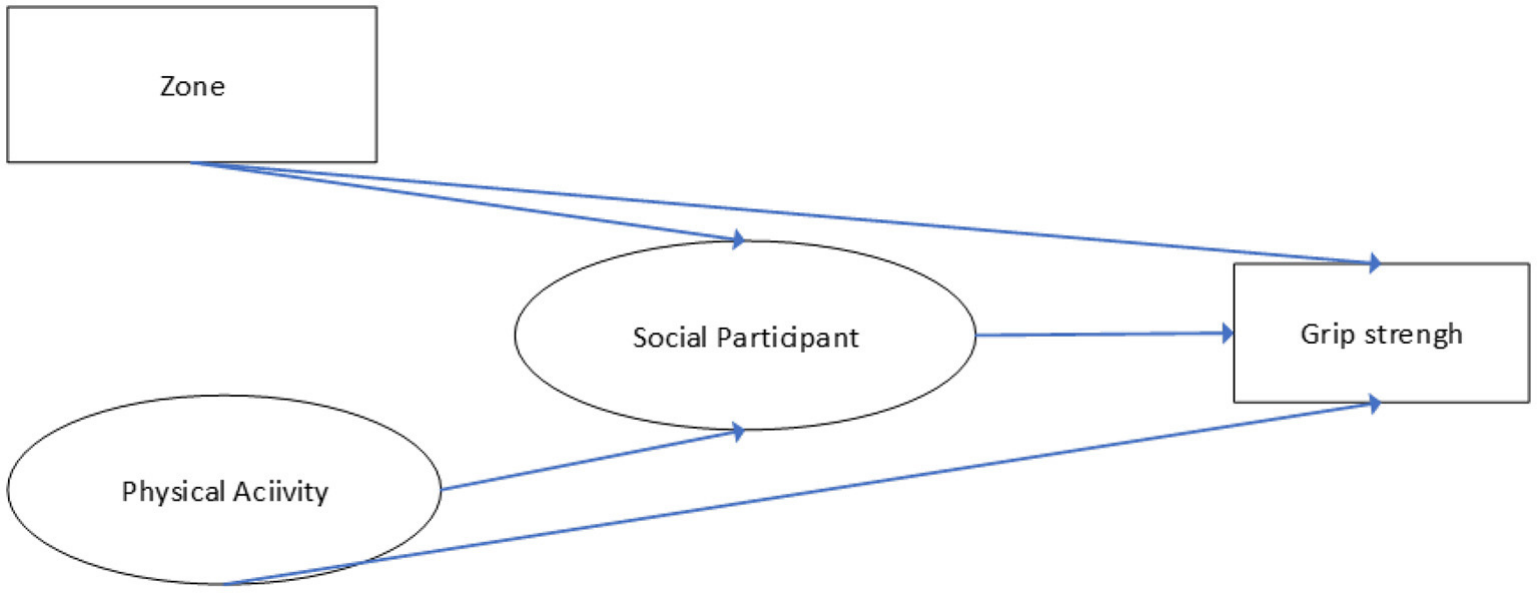
Construction of the conceptual model.

Only a few articles in the literature, such as Celis-Morales et al. (43) provided specific measures of the impact of PA and SA on grip strength. Therefore, this article proposess a structural equation model and performs factor analysis of latent variables to correct possible measurement errors. Moreover, this article classified essential variables, such as SP, PA, and Zone (Differences in residential communities) (Figure 1).

## Method

### Data

This study's database comes from the fourth wave of follow-up surveys conducted in 2015 for the CHARLS. The CHARLS is a national survey that provides comprehensive and high-quality data on population background, family characteristics, health behaviors, and Chinese citizens' status and retirement information (54). Using probability proportional to size (PPS) sampling and computer-assisted personal interview (CAPI) technology, interviews were conducted with middle-aged and elderly respondents in 450 communities in 150 counties in 28 provinces. The Biomedical Ethics Review Committee of Peking University approved the study (IRB00001052–11015). The baseline survey (the first wave) was conducted in 2011; among the community residents who participated in the baseline survey, 20,543 participated in 2015. To study the factors influencing the grip strength of the elderly population, this article limited the sample of respondents to those over 50 years old. This article included 15871 respondents (7807 male, 8064 female) who participated in the fourth wave of the study. More details can be found from http://charls.pku.edu.cn/Public/ashelf/public/uploads/document/2018-charls-wave4/application/CHARLS_2018_Users_Guide.pdf (55, 56).

### Measurement

This study used measurement items for some constructs from the relevant literature. This article chose some items to assess PA, SP, and respondents' relationships with their adult children. Some items also collected demographic information about respondents, such as biomarkers, age, expenditure, and smoking and drinking behavior. All items were measured (converted to ordinal or quasi-ordinal scales) on a 7-point or 6-point scale from low to high due to the comparability of quantity or frequency.

#### Dependent Variable

##### Grip Strength

The grip strength index uses the average values for the left and right hands from the second test. The measurement method lets interviewees “Stand, hold the dynamometer at a right angle and squeeze the handle for a few seconds, then Conduct the hand strength measurements (unit: kilogram).” This article did not use the grip strength-to-weight ratio because many studies have indicated that the correlation between grip strength and weight is not very strong. The grip strength-to-weight ratio was entered into the model as an explanatory variable. The result was not much different in terms of the direction of the odds ratios.

#### Independent Variables

##### Differences in Residential Communities (Zone)

The population density in China's urban and rural areas gradually decreases from metropolises, provincial administrative centers, suburbs of large cities, general prefecture-level cities, county-level cities, and townships from 4,000, 2,000, 1,000, and 500 peoples/km^2^, respectively (57). Additionally, some studies have investigated the development of the suburbs of large cities (58, 59). The population density has a decisive effect on interpersonal communication based on the CHARLS data; regarding interpersonal communication methods, in China, the urban-rural fringe is more active and inclusive in relation to activities among the elderly- population than urban areas. Due to the non-enclosed residential areas and semi-urbanized (preserving more rural lifestyle habits) habits, towns (county-level cities) also have similar characteristics as the urban-rural fringe of a big city (the surrounding rural population settles in the city). According to this idea, this article found that the first type of interpersonal communication is entirely related to density (interaction with friends declined in recent years). The second activity (playing mah-jong, chess, or cards) is related to communication habits. Thus, this article made slight adjustments to the ranking order of the first three types of residential areas based on the original questionnaire's order according to the population density and the frequency of participation in mah-jong card games (Mah-jongg is a traditional Chinese game similar to playing cards and it has some gambling properties) and tested the ranking with the Pearson correlation coefficient. In other words, different kinds of regional rankings respect the original questionnaire, which is based on the dimension of population density. However, it is also adjusted according to the frequency of social activities, which aligns with the original intention of defining the community based on population density and social activities.

Residents of large cities come from different backgrounds, but they have a low level of intimacy. Except for these large cities, most of China's cities are predominantly populated by migrants from nearby rural areas or small cities. Therefore, there are no significant differences in residents' backgrounds in these cities. Most of these cities have rural Chinese social characteristics, which means that the community is not composed of sporadic long-distance immigrants; even if there are some long-distance immigrants, they cluster in groups. Because China's most significant cultural differences are not due to migrating from long distances but rather to the urban-rural binary, community residents who moved to the community from a rural area are not culturally diverse from their neighbors (60). In other respects, compared with their urban counterparts, rural residents have a limited number of social networks, but these networks are reliable and stable (52, 61–63).

##### Physical Activity

The original questionnaire distinguishes high-intensity PA, medium-intensity PA, and low-intensity PA. Since singing and dancing for leisure and entertainment have PA characteristics, singing and dancing are included as low-intensity PA as a supplementary indicator for intense PA.

#### Mediating Variable: Social Activity

Douglas et al. (22) recommended using the classification standards for broad social participation, semiformal social support, and formal social support. According to his definition, the government provides China's formal social support, but it is just at the beginning stage, So the previous data is insufficient to be used. Therefore, this article divides social activities into general social activities, helping others, and joining social groups instead of using such a classification.

#### Potential Confounding Variables

Potential confounding variables were considered the relationship with adult children, smoking, upper-body pain, and basic expenditure (Table 1).

**Table 1:** Description of the main variables and descriptive statistics of the sample.

				**Total sample**		**Male**		**Female**	
**Topic of questionnaire item**	**Question content**	**Variable name in model**	**Recode/redifinite**	**Mean**	**Std.Dev**.	**Mean**	**Std.Dev**.	**Mean**	**Std. Dev**.
				***N*** = **15,871**		***N*** = **7,807**		***N*** = **8,064**	
Grip strength	gc003–gc004 Conduct the hand strength measurements	GP	Grip1 = (qc003 + qc004)/2 (The average of the left and right hand)	4.067	2.063	4.733	2.282	3.422	1.577
Gender	a000_w2_3	GENDER							
Area of residence	1. Main city zone; 2. Combination zone between urban and rural areas; 3. Town center;4. ZhenXiang area 5. special area 6. Township central 7. Village	AREA	Recode zone (1 = 3) (2 = 1) (3 = 2) (4 = 6) (6 =4), this article did not know the 5 special area, but there only 53 samples.so this article keep the code.	5.819	1.995	5.823	1.999	5.815	1.992
									
Age	ba004_w3_1 birth year	AGE	Age = 2015-ba004_w3_1	62.821	9.098	62.893	8.933	62.751	9.254
High PA	da051_1:Vigorous activities that make you breathe much harder than normal and may include heavy lifting, digging, plowing, aerobics, fast bicycling, and cycling with a heavy load	HPA	Use days (from da052)* hours (from da054 to da055). Recode da054 and da055 which <30 min, more than 30 min <2 h, more than 2 <4 h and more than 4 h to 0.5, 1.5, 3, and 5.then use the hours value to mulplus da052. Also can see the formular in Lpa	4.125	1.913	4.155	1.910	4.096	1.916
Moderate PA	da051_2:Moderate physical activity including carrying light loads, bicycling at a regular pace, or mopping the floor	MPA		2.384	2.404	2.307	2.369	2.460	2.436
Low PA	da051_3:Physical activity including walking to travel and any other walking that you might do solely for recreation or sport exercise, or leisure	LPA	Lpa = da052_3*flu (recode da054 and 055) + da056_4*da057 (recoded)*2	2.755	2.371	2.760	2.372	2.750	2.370
Singing and dancing	See SA								
Frequency	da052_(1–3):During a usual week, on how many days did you do (1-3) for at least 10 min?		Singing and dancing are 2 h each time by default, so as to unify the dimension with physical activity	2.734	1.072	2.654	0.936	2.812	1.185
	da054 and da055:How much time did you usually spend doing (1-3) on one of those days?								
SA	da056_(s1 ~ s11):Have you done any of these activities in the last month?	SA1	Sa1 = [Q(question)1*da057_1_ + Q2*da057_2_ + Q8* da057_8_ + Q9 da057_9_ + Q10*da057_10_ + Q11*da057_11_]*2	3.077	2.411	3.179	2.433	2.978	2.385
	1. Interacted with friends; 2. Played mah-jong, played chess, played cards, or gone to a community club; 3. Provided help to family, friends, or neighbors who do not live with you; 4. Gone to a sport, social, or other kind of club; 5. Taken part in a community-related organization; 6. Done voluntary or charity work; 7. Cared for a sick or disabled adult; 8. Attended an educational or training course; 9. Stock investment; 10. Used the Internet; 11. Other	SA2	Sa2 = (Q4*da057_4_ + Q5*da057_5_)*2	3.893	1.092	3.737	1.111	2.807	1.074
	Cf003 and cf004:Approximately how many weeks and how many hours per week did you spend last year taking care of this child's children? cf003 and cf004	SA3	Sa3= Q3*da057_3 + Q6*da057_6 + Q7*da057_6 + Taking care of grandchildren + taking care of the elderly	4.173	2.294	3.243	2.309	3.106	2.277
			Here, SA1, SA2, and SA3 are divided into general social activities, organized activities, and social participation (semi formal help and volunteers) by converting each activity time which include in da056 into 2 h						
			da057 the value was 1 to 3, from almost daily to not regularly. recode da057-1~11 (1–6) (2–3) (3–1)						
	CF005 cf006:Approximately how many weeks and how many hours per week did you yourself (or your spouse) spend last year taking care of your parents or parents-in-law								
	DA057: Frequency of activity in the last month 1. Almost daily; 2. Almost every week; 3. Not regularly								
Children Relation	cb053:Living near one's child	Chifar		3.511	2.357	3.662	2.358	3.366	2.347
	cb069:Child's income	CHInc		3.385	2.484	3.617	2.509	3.160	2.438
	cd003:Seeing children	SeeCh		3.506	2.451	3.666	2.444	3.352	2.448
	da042 on what part of your body do you feel pain? Please list all parts of body you are currently feeling pain	Up pain	Gen upain = da042s1 + da042s2 + da042s3 + da042s4 + da042s5 + da042s6 + da042s8 + da042s15 1. Head (Headache); 2. Shoulder; 3. Arm; 4. Wrist; 5. Fingers; 6. Chest; 8. Back; 15. Neck						
Smoking	DA063 in 1 day, about how many cigarettes do/did you consume?	Smoking		2.461	2.335	3.689	2.600	1.271	1.144
Basic expenditure Rate	GE007 among it, how much did your household spend on eating out?	BE	basicexp1 = ge007 ++ ge009_2 + ge010_11 + ge009_3 + ge010_1 + ge010_3 + ge010_11 [eating out; Utilities: Water and electricity; Fuels (including gas, coal, etc.); Clothing and bedding; Heating(centrally heated); Property management fees]	3.144	2.556	3.639	2.638	2.666	2.378
	GE008 among it, how much did your household spend on alcohol		Texpend = sum (ge007, ge008, ge009(1 ~ 7), ge010*(1 ~ 5), ge010(7 ~1 3))						
	GE009 what was the expenditure in the last month for your household for the following items		geneindex1 = basicexp1/texpend						
	GE010 in the last year how much did your household spend on the following items?		The numerator and denominator don't include medical expenses	3.144	2.556	3.639	2.638	2.666	2.378
Upper-Body pain	da042 on what part of your body do you feel pain? Please list all parts of body you are currently feeling pain	Up pain	Gen upain = da042s1 + da042s2 + da042s3 + da042s4 + da042s5 + da042s6 + da042s8 + da042s15 1. Head (Headache); 2. Shoulder; 3. Arm; 4. Wrist; 5. Fingers; 6. Chest; 8. Back; 15. Neck	1.563	1.262	1.358	0.998	1.761	1.446

*GP, Grip strength; GENDER, gender; AREA, Area of residence; AGE, age; HPA, High physical activity; MPA, Moderate physical activity; LPA, Low physical activity; SA, Social activity; Chifar, Living near one's children; CHInc, Children's income; SeeCh, Seeing children; Up pain, Upper-body pain; BE, Basic expenditure rate*.

### Analysis

Descriptive statistical analysis and structural equation modeling (SEM) were used in the study. SEM model is often used in such databases with many ordered variables (64–66). The structural equation model is advantageous for quantitative analysis and group comparison of multivariate interaction effects and can help correct measurement errors. Therefore, this article used a structural equation model to analyze the relationship among PA, community differences, social participation, family relations, and grip strength. All variables had good discrimination and were suitable for SEM. Furthermore, the sample size was larger than 1,000, and samples of this size can generally be considered to follow a normal distribution.

### Confirmatory Factor Analysis

CFA was used to measure PA, relationships with adult children, and SP. According to the results, the composite reliability and validity coefficients in all measurement models were high, indicating that all measurement models had excellent reliability and validity and were suitable for SEM analysis.

Regarding the fit indexes of the entire sample model, the root mean square error of approximation (RMSEA) was 0.075, the ratio of chi-square to the degrees of freedom (X2/DF) was 4.902, the comparative fit index (CFI) was 0.912, the Tucker-Lewis index (TLI) was 0.903, and the standardized root mean square residual (SRMR) was 0.059; these fit indexes indicated that the models had a good fit, and they are shown in Table 2.

**Table 2:** Item reliability, composite reliability, convergent validity, and discriminant validity.

**Dimension**	**Items**	**Item reliability**	**Composite reliability**	**Convergent validity**	**Discriminant validity**		
		**Standard Loading**	**CR**	**Average Variance Extracted**	**Physical Activity**	**Social Activity**	**FAMILY**
PA	3	0.844–0.993	0.962	0.894	**0.946**		
SA	3	0.505–0.869	0.746	0.506	0.046	**0.711**	
CR	3	0.488–1.322	0.913	0.806	−0.047	−0.023	**0.898**

*PA, Physical activity; CR, Children relation; SA, Social activity; AVE, Average variance extracted; PHYAC, Physical activity; SOCAC, Social activity; DIM, Dimension; Std. loading, Standard Loading*.

## Results

### Descriptive Statistics of the Sample

The total sample size was 15,871, including 7,807 males and 8,064 females. The average grip strength of males was significantly higher than that of females. The average community type score was 5.82, indicating that most of the respondents lived in non-large cities. Men had moderate PA levels that were slightly lower than those of women. Singing and dancing activities were more common among women than men. Women had higher levels of overall SA than men, but men engaged in more general social participation than women. Female upper-body pain was more common than male upper-body pain.

### Composite Reliability and Convergent Validity

The CR values of the measured variables ranged from 0.746 to 0.962; a CR value above 0.6 is recommended by Bagozzi and Yi (67) and Fornell and Larcker (68); thus, the research variables were within the acceptable range. Finally, this article measured validity according to convergent and discriminant validity as proposed by Anderson and Gerbing (69). Table 2 shows that each measured variable reached significance. The AVE values ranged from 0.506 to 0.894; a value above 0.5 is recommended by; thus, the variables were all accepted. Therefore, the measurement model had good convergent validity. This result indicates that all measured models had excellent reliability and validity and were suitable for the SEM analysis.

Among the three items, the reliability (assessed by the squared multiple correlations, SMC) was the highest for PA. The validity of the general social interaction dimension of SA was relatively low but was still above 0.5. The reliability of children's income and frequency of parent-child interaction was not very strong but was still higher than the standard. The item validity values were 0.946 (PA), 0.711 (SP), and 0.898 (CR), which were all >0.7, indicating that the items had an excellent explanatory ability for the selected dimension.

### Comparison of the Model Paths by Gender and Covariance Coefficient and Fit Indexes for the Whole Sample

**Figure 2:**
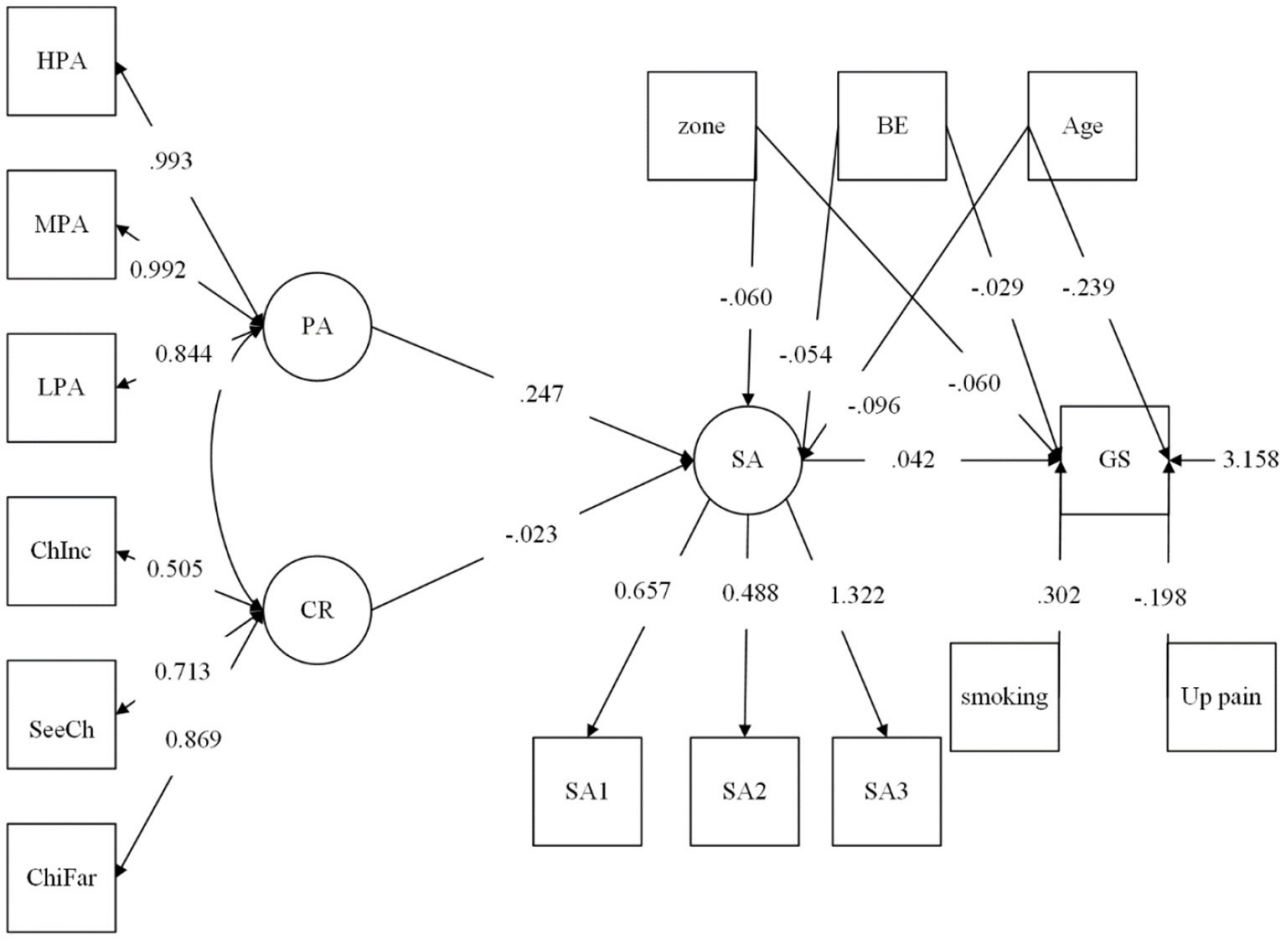
Standardized coefficients for the full sample model. GP, Grip strength; GENDER, gender; AREA, Area of residence; AGE, age; HPA, High physical activity; MPA, Moderate physical activity; LPA, Low physical activity; SA, Social activity; Chifar, Living near one's children; CHInc, Children's income; SeeCh, Seeing children; Up pain, Upper-body pain; BE, Basic expenditure rate.

When mediating variables are present in a model, the relationship between independent and dependent variables should be expressed as total, direct, and indirect effects. Table 3 and Figure 2 show the results of the entire sample model.

**Table 3:** Comparison of the model paths by age and the odds ratios and fit indexes.

**Dependent variable**	**Whole sample**			**Subgroup**			
**Grip strength**	**Coefficient**	***Z***			**OR**	***Z***	
Total effects from PA to GRIP	0.01	3.894	***	Male	0.048	3.355	**
Specific indirect effect through SA to GRIP	0.01	3.894	***		0.005	1.797	*
DIRECT EFFECT					0.042	2.901	***
Total effects from PA to GRIP				Female	0.016	3.273	***
Specific indirect effect through SA to GRIP					0.016	3.273	***
DIRECT EFFECT					−0.002	−0.118	
Total effects from ZONE to GRIP	−0.062	−5.795	***	Male	−0.091	−6.216	***
Specific indirect effect through zone to GRIP	−0.002	−3.115	***		−0.002	−1.771	*
Direct effect	−0.06	−5.564	***		−0.089	−6.056	***
Total effects from ZONE to GRIP				Female	−0.066	−4.174	***
Specific indirect effect through SA to GRIP					−0.003	−2.508	**
Direct effect					−0.063	−3.976	***
Total effects from AGE to GRIP	−0.243	−22.861	***	Male	−0.43	−31.473	***
Specific indirect effect through age to GRIP	−0.004	−3.535	***		−0.005	−2.4	**
Direct effect	−0.239	−22.418	***		−0.425	−30.72	***
Total effects from AGE to GRIP				Female	−0.35	−23.073	***
Specific indirect effect through SA to GRIP					−0.003	−2.227	**
Direct effect					−0.347	−22.857	***
Total effects from ENGLE to GRIP	−0.031	−2.864	***	Male	−0.017	−1.974	**
Specific indirect effect through expenditure level to GRIP	−0.002	−2.993	***		−0.002	−1.792	*
Direct effect	−0.029	−2.653	***		−0.015	−1.832	*
Total effects from ENGEL to GRIP				Female	−0.035	−3.264	***
Specific indirect effect through SA to GRIP					−0.003	−2.397	**
Direct effect					−0.032	−3.088	***
Upper-Body pain	−0.198	−18.877	***	Male	−0.14	−8.517	***
				Female	−0.141	−8.736	***
Smoking	0.030	28.23	***	Male	0.002	0.147	
				Female	0.02	2.489	**
SP	0.042	3.949	***	Male	0.037	2.456	***
				Female	0.051	3.362	***
SA with children	−0.023	−2.012	**	Male	−0.002	0.906	
				Female	−0.045	−2.539	**
Fit indexes for model (1)	ML X2	DF	X2/DF	CFI	TLI	RMSEA	SRMR
	598.000	122.000	4.902	0.912	0.903	0.075	0.059

***p < 0.01; **p < 0.05; *p < 0.1.

*GRIP, Grip Strength; GENDER, gender; AGE, age; PA, Physical Activity; SA, Social Activity; Chifar, Living near one's children; CHInc, Children's income; SeeCh, Seeing children; BE, Basic expenditure Rate; ZONE, Differences in residential communities; ENGLE, Engel's Coefficient*.

The correlation between the relationship with adult children and SA was significant (−0.023), and the correlation between smoking and grip strength was −0.302. The correlations of upper-body pain and grip strength (−0.198), SA and PA (0.247), and SA and grip strength (0.042) were all significant.

### Group Analysis

According to the various direct and indirect effects for the male and female groups, the total effect of PA on grip strength was significant for both the male group (0.007) and the female group (0.016). However, in terms of the effect of PA on grip strength, the direct effect in the male group was significant, and the direct effect in the female group was not significant, indicating that PA did not directly generate changes in grip strength. In contrast, social activity significantly affected grip strength in both the male and female groups. The effects of community differences on grip strength were significant in terms of both the total effect (−0.062) and the direct effect (−0.06) (but if this article used the 95% confidence interval, then the relationship between community differences and social participation was not significant in the male group). Thus, community difference was related to grip strength using the 95% confidence interval in the female group. Residents of areas with lower population density had lower SA levels, and residents of large cities had lower SA levels than residents of the urban-rural fringe and county-level cities. This finding shows that behavioral patterns based on an agricultural society and population density influence social participation. Notably, the direct effect of community differentiation (−0.06) on grip strength was more significant than the indirect effect (−0.002). This finding shows that community differences affect grip strength and social participation and may also affect grip strength through medical facilities and other reasons that are not yet clear. This discussion indicates a direction for follow-up research, but some studies have argued that this effect is not related to the question of whether infrastructure helps increase PA (70). The direct effect of individual age changes on grip strength (−0.243) was more significant than the indirect effect on grip strength through social participation (−0.004), indicating that the age effect of weakening grip strength is much higher than that of social participation.

Essential expenditure also affected grip strength, indicating that nutrition or eating habits also affect grip strength. The indirect effect of basic expenditure rate on grip strength through social interaction (−0.002) was much smaller than the direct effect on grip strength (−0.031), which shows that there was no significant correlation coefficient between social identity and grip strength (the direct correlation coefficient between expenditure and grip strength was much stronger than the relationship between expenditure level and social identity). See Table 3 for details. Therefore, Hypothesis A, B, C, and D are all accepted in the entire sample. However, in the male group, Hypotheses A is not accepted (see Table 4).

**Table 4:** Hypothesis test results.

**Hypotheses**	**Results (Accepted or not)**		
	**All samples**	**Male**	**Female**
HA: Moderate PA has a positive effect on grip strength	Yes	Yes	No
HB: Positive types of SA ameliorate the decline in the grip strength of elderly people	Yes	Yes	Yes
HC: The combination of population density and neighborhood relations is positively correlated with SP	Yes	Yes	Yes
HD: Living standard is positively related to grip strength	Yes	Yes	Yes

*HA, Hypothesis A; HB, Hypothesis B; HC, Hypothesis C; HD, Hypothesis*.

## Discussion

This article discussed the influence of SA and PA on grip strength based on a sample from the CHARLS, analyzed the factors that affect social interaction and PA and the interaction between the two variables, and finally examined these effects within structural equation models of the effect on the change in grip strength.

### Regional Differences

Some studies have discussed the differences in behavior and health status between urban and rural areas in developed countries (71). Some papers studied the differences in social life, including health, between urban and rural areas in developing countries (72). However, it is not mentioned that the difference in interaction patterns between urban and rural areas in developed and developing countries is due to rural living habits. Especially in health research, various urban and rural community forms are divided in detail according to different population densities and behavior patterns (rather than simply separating into rural and urban areas). There are very few studies like that, concentrated on spatial economic analysis (73). As for the SEM of community differences (between urban and rural areas divided by population density and behavior)—social activities—health, it has not been found in the open literature. According to the empirical analysis results of this study, Chinese elderly people who visit their families and friends, play card games, and dance have more obvious correlations with grip strength. Result also shows that the community difference caused by the frequency of social activities can better explain the relationship between the grip strength of the elderly and the community difference than the difference caused by the simple population density. The reason behind this difference may be caused by different ways of interpersonal communication.

### Age

The efforts of non- intervening exogenous variables such as age on grip strength can be found to be partially intervenable, rather than only be used as control variables (74, 75). However, the relationship between age growth and social activities is not so significant. This shows that it is a way to improve grip strength by intervening the correlation coefficient between age and social activities to increase beneficial social activities to delay the reduction of grip strength, although the effect is limited. Similarly, the effect of marital status on grip strength can also be decomposed by SEM.

### Social Activities

Quirke et al. (76) believe that the impact of for the taking care of grandchildren on other social activities and self-rated well-being is complex. This study does not discuss the competitive relationship between the taking care of grandchildren and other social activities but expands the scope of social activities to consider the impact on grip strength. There is no such practice in other articles on grip strength and social activities (77, 78).

### Physical Activity

The direct effect of PA on grip strength was significantly negative, and the effect of PA on promoting SA was significant. This result is similar to the idea of, Kuh et al. (35) and Jenkin et al. (79). Suppose grip strength is an essential indicator or even the only indicator of health (other than self-rated health) among elderly people. In that case, the strategy of increasing physical exercise among middle-aged and older people may be an option that needs to be more carefully reviewed. In other words, we need to pay more attention to the conclusion made by Yu et al. (78) that the change of grip strength can be improved by increasing social activities rather than physical activities.

### Further Work

Areas for further work include the identification of more variables in the questionnaire that better describe community differences and the use of more biomarkers to indicate weakness to allow the analysis of environmental differences in more detail as well as physical exercise, social participation, and the relationship between specific indicators of the health of elderly people. In addition, in the future, it will also be necessary to use longitudinal data to reflect the cause-and-effect relationship when the sample size and sample periods are sufficient.

The relationship between cognition and SA was not examined in this study because cognition and SA have two-way interactions, and the relationship between grip strength and other variables such as cognition may also be two-way due to the complexity of the problem, rendering these variables more suitable for a separate research project.

The relationship between regional differences and social relations, as well as the relationship between regional differences and grip strength, may not be limited to differences in economic conditions, environmental conditions, and SP; they may also involve other aspects of urban and rural differences in China, such as long-term physical labor vs. grip strength and differences in the nutritional structure during adolescence. Therefore, a study making comparisons based on regional differences to examine the relationship between grip strength and regional differences through social interaction may have the problem of missing variables.

The social items on the questionnaire could be classified as general social interactions or different types of SP. However, according to this classification, the sample size for some types was too small, which affected the significance of the statistical results. In other words, due to the lack of data samples, such classifications could not be tested extensively. Therefore, our results suggest that general SA positively affects grip strength, and there is no way to distinguish between the differences among different kinds of activities.

The grip strength-to-weight ratio was not used because the correlation between grip strength and weight is not strong enough, and adding weight may complicate the problem.

### Limitations

There are some limitations to this study. First, a more detailed analysis by age group is recommended; the sample used focused on the 50–69-year-old population, and the elderly population over 70 years old was much smaller than the younger elderly sample. Since the sample sizes of each age group were so different, the age groups were ignored. Second, regarding whether to use confirmatory factor analysis, there are also many works on the health of elderly people that do not use latent variables but directly use all measured variables. We also tried to construct a structural equation model with a single measurable indicator, and the general conclusion was similar to the result using factor analysis. The structural equation model, including factor analysis, was used to correct the measurement errors at the cost of not performing more detailed analyses.

## Conclusion

The contribution of this study lies in (1) establishing a structural equation model including community differences, social activities, and specific biomarkers; (2) comparing the direct and intermediate effects of age and gender on grip strength, indicating that the effects of age and gender on grip strength can be intervened; (3) based on the difference of population density and social habits, we modified the scoring criteria of community types and ranked them, to reveal more insights into the difference of social activities between urban and rural areas on physical health indicators; (4) including the occasional care of grandchildren and elderly parents into the scope of active social activities. The positive effect of positive social activities on grip strength was further confirmed.

This study enriches the research of grip strength in the elderly population, increases the complexity of the understanding of the intermediate effects, and indicates that the influence of non-interventional exogenous variables such as age and gender on grip strength is partially endogenous (intervenable) through the separation of the intermediate effects and the comparison of the indirect and direct effects. The community differentiation approach differs from that in the original questionnaire, revealing the characteristics of the SA of elderly residents in urban and rural areas in China and the impact of these differences on grip strength.

The inspiration for this study to formulate policies and support for the elderly population lies in the importance of community differentiation. In contrast to common perception, the community interactions of elderly residents living at the junction of urban and rural areas are higher than those of elderly residents in large urban centers. This finding indicates that some behavioral patterns associated with agricultural society are beneficial to the SA of elderly people. In addition, according to the coefficient of the effect of basic expenditure level on grip strength, improving quality of life requires unremitting efforts to improve physical health. Finally, the importance of elderly people's interactions with their adult children for their health in East Asian countries needs to be emphasized. Encouraging childbirth is a policy option as circumstances permit.

## Data Availability Statement

The datasets presented in this study can be found in online repositories. The names of the repository/repositories and accession number(s) can be found in the article/Supplementary Material.

## Ethics Statement

Ethical review and approval was not required for the study on human participants in accordance with the local legislation and institutional requirements. The patients/participants provided their written informed consent to participate in this study. Ethical approval for all the CHARLS waves was granted from the Institutional Review Board at Peking University. The IRB approval number for the main household survey, including anthropometrics, is IRB00001052-11015; the IRB approval number for biomarker collection, was IRB00001052-11014.

## Author Contributions

All authors listed have made a substantial, direct, and intellectual contribution to the work and approved it for publication.

## Funding

This research was funded by the Project of the Natural Science Foundation of Liaoning Province, China (Grant No. 71490735) and Social Science Fund of Liaoning Province, China (Grant No. L19BGL035).

## Conflict of Interest

The authors declare that the research was conducted in the absence of any commercial or financial relationships that could be construed as a potential conflict of interest.

## Publisher's Note

All claims expressed in this article are solely those of the authors and do not necessarily represent those of their affiliated organizations, or those of the publisher, the editors and the reviewers. Any product that may be evaluated in this article, or claim that may be made by its manufacturer, is not guaranteed or endorsed by the publisher.

## Abbreviations

PA: Physical activity
SP: Social participation
RL: Relation with adult children
SPA: Slight physical activity
GSP: General social participation
ISS: Informed social support
GS: Grip strength.
